# Supplementary material for: Conformational dynamics of the membrane-anchored foldase LipH from Pseudomonas aeruginosa governs recognition and release of its client lipase
Source: J Biol Chem. 2026 Jun 19;302(8):113266. doi: 10.1016/j.jbc.2026.113266 (PMC13381987; doi:10.1016/j.jbc.2026.113266)
Supplement: Supplementary Material [file mmc1.pdf]

Conformational dynamics of the membrane-anchored foldase LipH from *Pseudomonas aeruginosa* governs recognition and release of its client lipase

Max Busch, Jennifer Loschwitz, Athanasios Papadopoulos, Jens Reiners, Wieland Steinchen, Vincenzo Calvagna, Sander H.J. Smits, Karl-Erich Jaeger, Alexej Kedrov

Supporting information

Table of Contents

Figure S1. Conserved organization of the foldase:lipase complex. S2
Figure S2. Gating-associated dynamics of *P. aeruginosa* LipA. S2
Figure S3. LipH architecture and design of the studied constructs. S3
Figure S4. LipH retains its secondary structure over the course of molecular dynamics simulations. S4
Figure S5. Conformational dynamics of LipH at 150 mM NaCl. S5
Figure S6. Conformational dynamics of LipH at 25 mM NaCl. S6
Figure S7. Maps of intramolecular contacts within LipH^{FL}. S7
Figure S8. LipH^{Chap} localization along molecular dynamics simulations. S7
Figure S9. LipH^{VD}:membrane interactions at different ionic strengths. S8
Figure S10. LipH interactions with the membrane interface. S9
Figure S11. Small-angle X-ray scattering analysis of LipH^{VD}. S10
Figure S12. SEC-MALS of the full-length LipH in DDM and Cymal-6 micelles. S11
Figure S13: *In vitro* analysis of LipH-mediated enzymatic activity of LipA. S11
Figure S14. Hydrogen/deuterium exchange mass spectrometry of LipA. S12
Figure S15. HDX behaviour of representative LipA peptides. S13
Figure S16. Hydrogen/deuterium exchange mass spectrometry of LipH^{Chap}. S14
Figure S17. HDX behaviour of representative LipH^{Chap} peptides. S15
Figure S18. Design of LipA fragments for studying interactions with LipH. S16
Figure S19. Co-elution assay to probe non-specific interactions of LipH. S16
Figure S20. Small-angle X-ray scattering analysis of the LipH^{VD}:LipA complex. S17
Figure S21. Small-angle X-ray scattering analysis of the LipH^{VD}:LipA$^{\Delta 81}$ complex. S18
Figure S22. AlphaFod3-based models of the membrane-anchored chaperones. S19
Figure S23. AlphaFold 3 analysis of a potential SecYEG:LipH^{FL} complex. S19
Figure S24. The electrostatic potential at the surface of the folded LipA. S20
Figure S25. Lipid-dependent activity of LipH^{FL}. S20
Table S1. Summary of SAXS data acquisition and analysis. S22
Table S2. Overview of data obtained by hydrogen/deuterium exchange mass spectrometry. S24
Supporting references S24

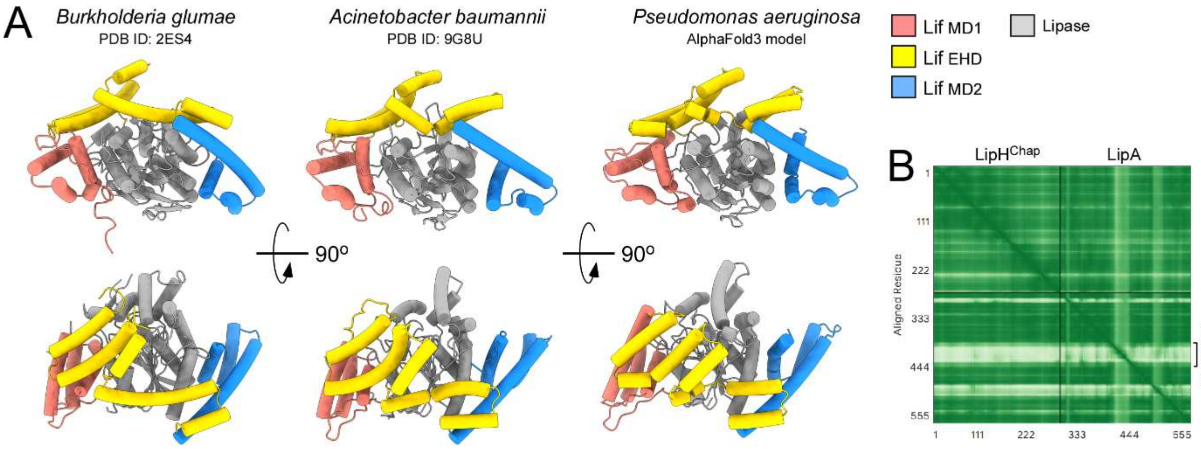

Figure S1. Conserved organization of the foldase:lipase complex.

(A) Crystal structures of lipases from *B. glumae* and *A. baumannii* bound to their cognate foldase chaperones (Lif's), in comparison to the AlphaFold3-derived model for the LipH^{Chap}:LipA complex of *P. aeruginosa* (excluding the N-terminal anchor/linker region; ipTM score 0.81).

(B) Predicted aligned error plot for the LipH^{Chap}:LipA complex of *P. aeruginosa.* The low-confidence region corresponds to the dynamic lid domain of LipA.

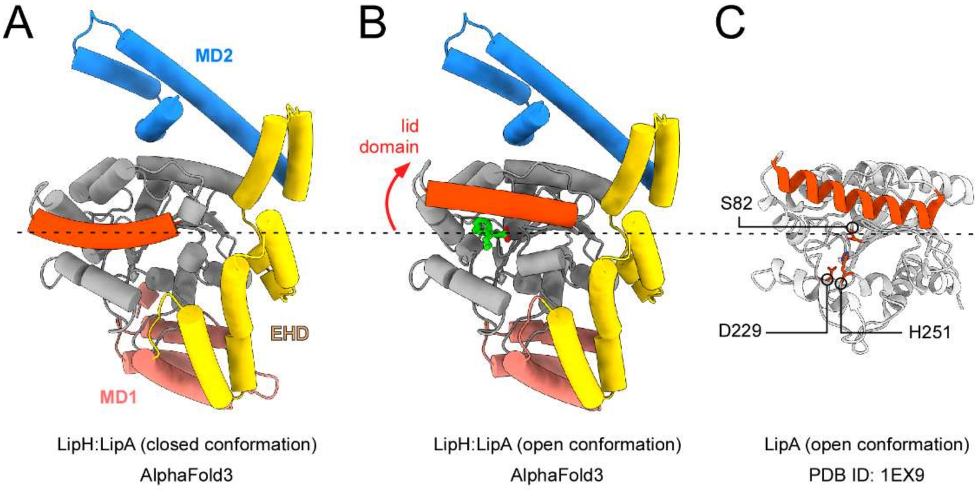

Figure S2. Gating-associated dynamics of *P. aeruginosa* LipA.

(A) AlphaFold3-derived model of the LipH:LipA complex. The gating helix 5 of LipA ("lid domain") is shown in red.

(B) AlphaFold3-derived model of the LipH:LipA complex in presence of an oleic acid molecule (green). Docking of the ligand is facilitated by a displacement of the lid domain, as indicated by the arrow.

(C) Crystal structure of *P. aeruginosa* LipA shows a displaced lid domain. The residues forming the catalytic triad are indicated.

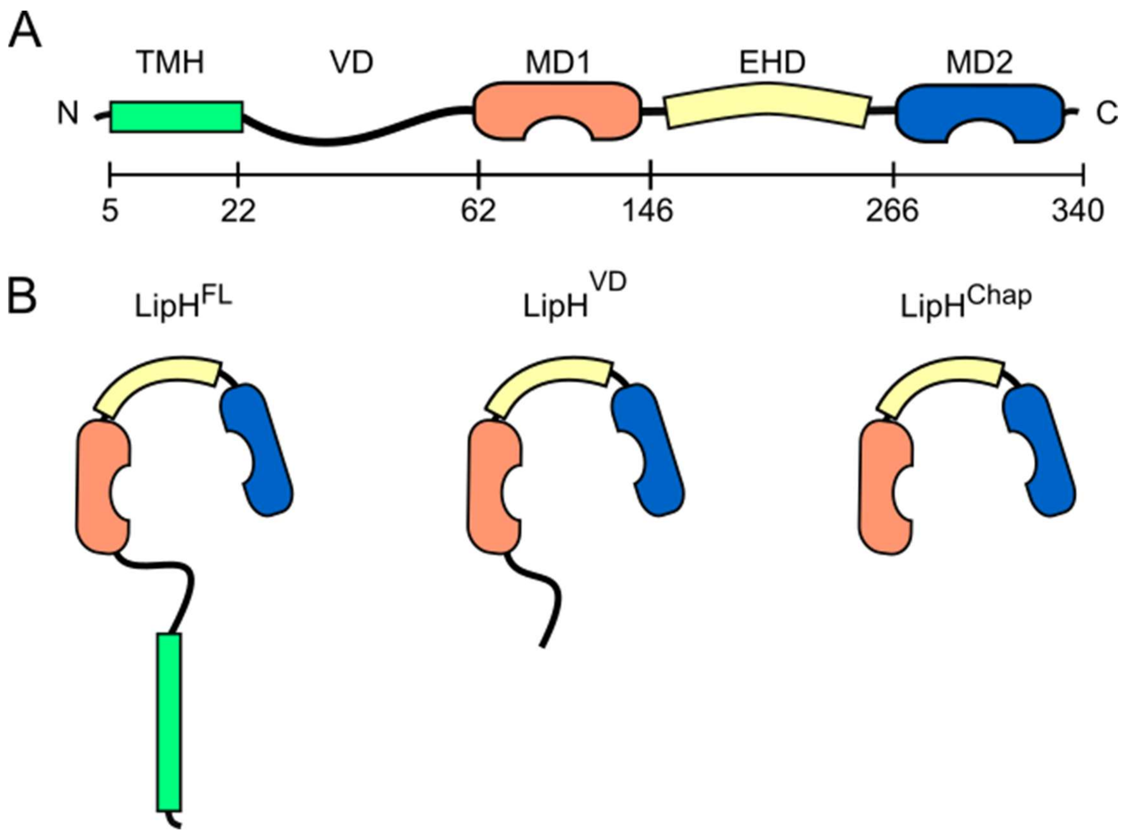

Figure S3. LipH architecture and design of the studied constructs.

(A) Organization of the full-length foldase LipH. The structural elements and their positions within the polypeptide chain (numbers in amino acids) are indicated.

(B) Schematic overview of the constructs employed in the study

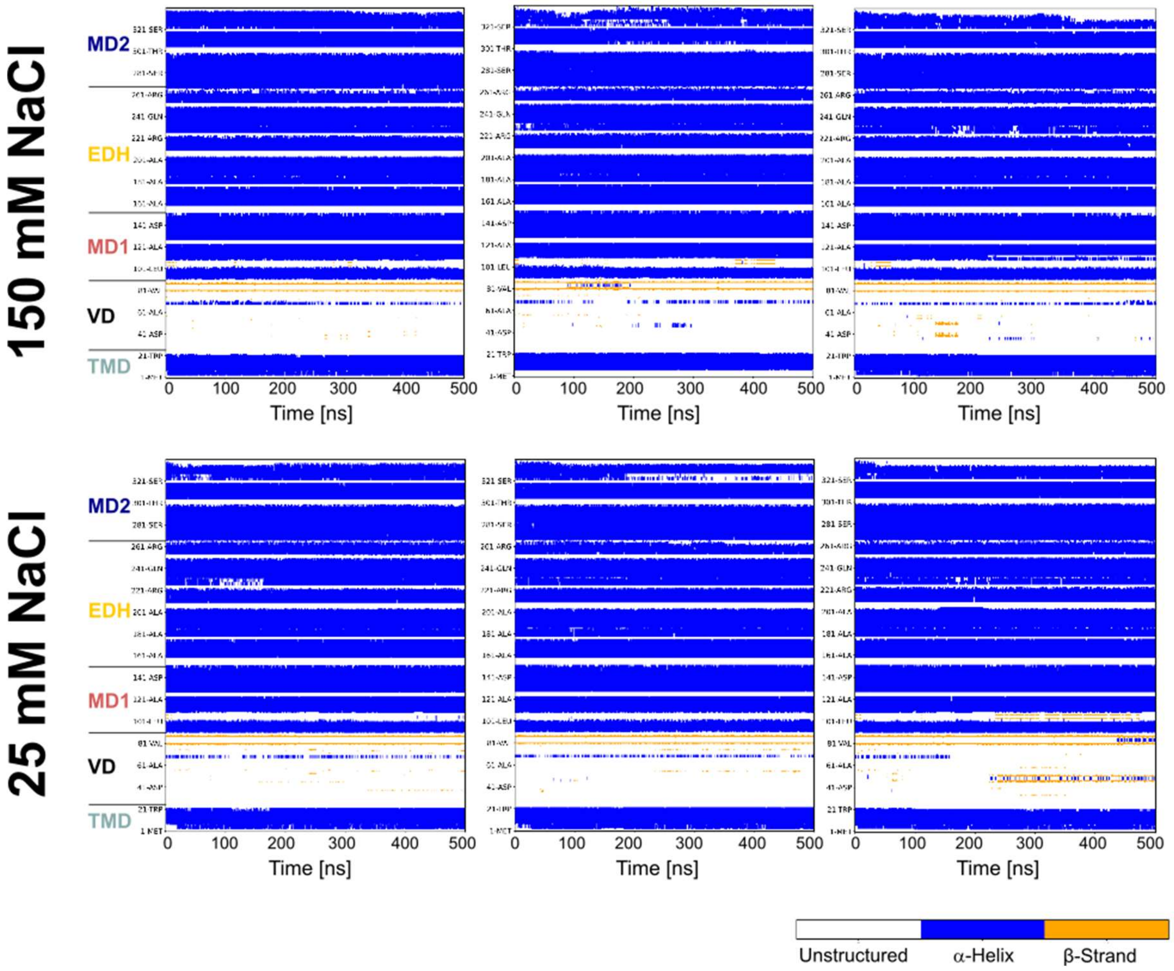

Figure S4. LipH retains its secondary structure over the course of molecular dynamics simulations. The color-coded secondary structure of LipH^{FL} along individual simulation courses at 150mM and 25 mM NaCl is plotted against the simulation time. The structural domains of LipH are indicated on the left.

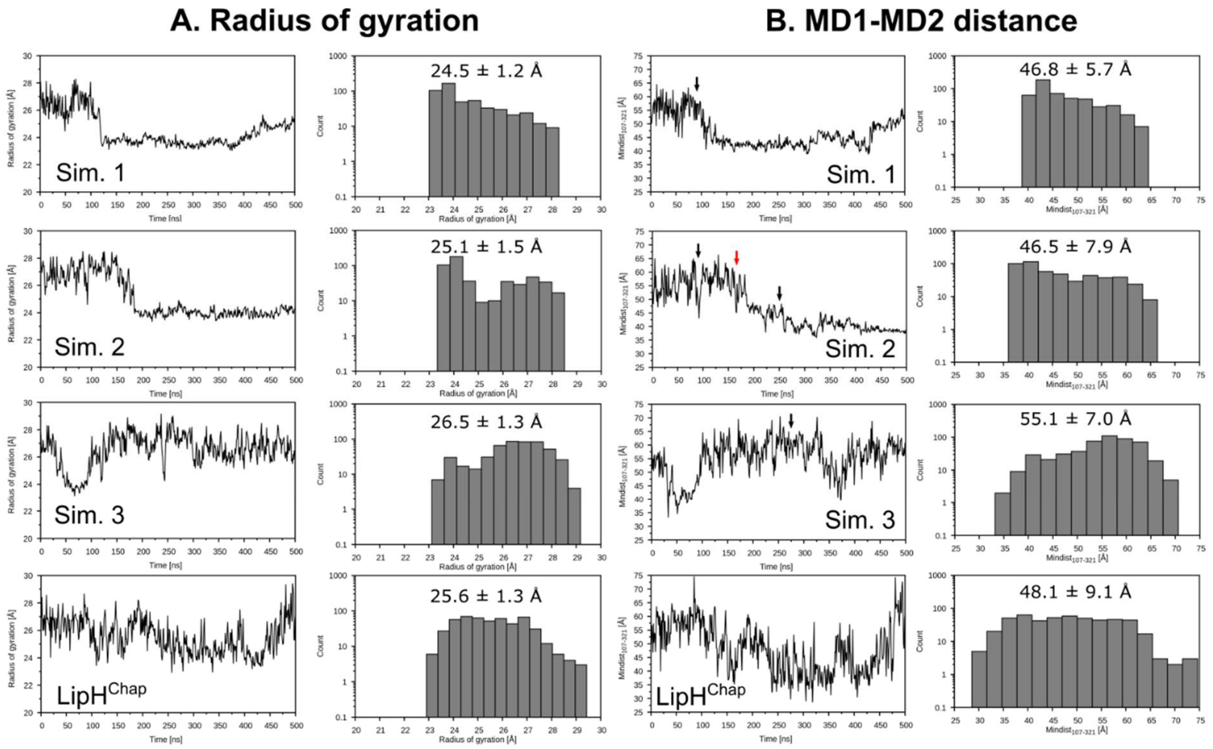

Figure S5. Conformational dynamics of LipH at 150 mM NaCl.

(A) Variations in the radius of gyration of the chaperoning domain within LipH^{FL} over the time course of individual simulations (left) and the corresponding distributions of the radius of gyration (right). The mean value ± standard deviations are indicated for each simulation. "LipH^{Chap}" corresponds to the LipH chaperoning domain simulated without the membrane anchor and VD.

(B) Variations in the distance between MD1 and MD2 domains within LipH^{FL} over the time course of individual simulations (left) and the corresponding distributions of the distance (right), with the mean value ± standard deviations indicated.

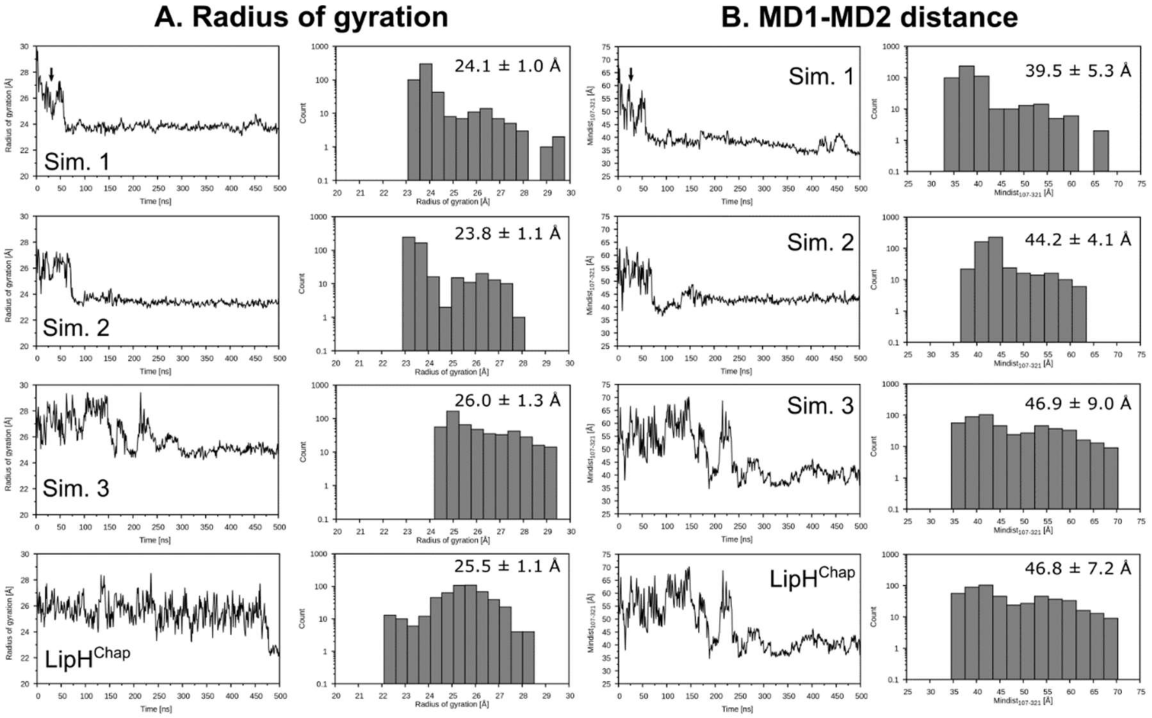

Figure S6. Conformational dynamics of LipH at 25 mM NaCl.

(A) Variations in the radius of gyration of the chaperoning domain within LipH^{FL} over the time course of individual simulations (left) and the corresponding distributions of the radius of gyration (right). The mean value ± standard deviations are indicated for each simulation. "LipH^{Chap}" corresponds to the LipH chaperoning domain simulated without the membrane anchor and VD.

(B) Variations in the distance between MD1 and MD2 domains within LipH^{FL} over the time course of individual simulations (left) and the corresponding distributions of the distance (right), with the mean value ± standard deviations indicated.

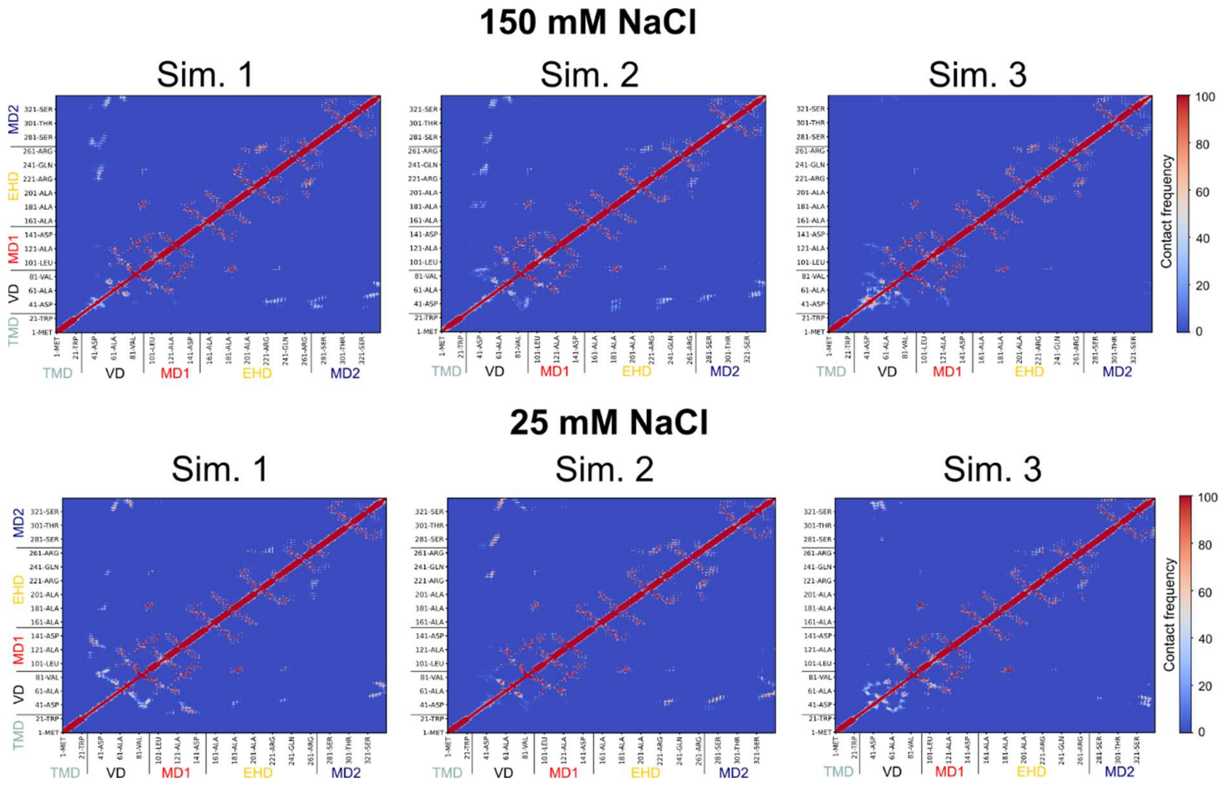

Figure S7. Maps of intramolecular contacts within LipH^{FL} . Over the course of individual simulations at 150 mM and 25 mM NaCl. The structural domains of LipH are indicated.

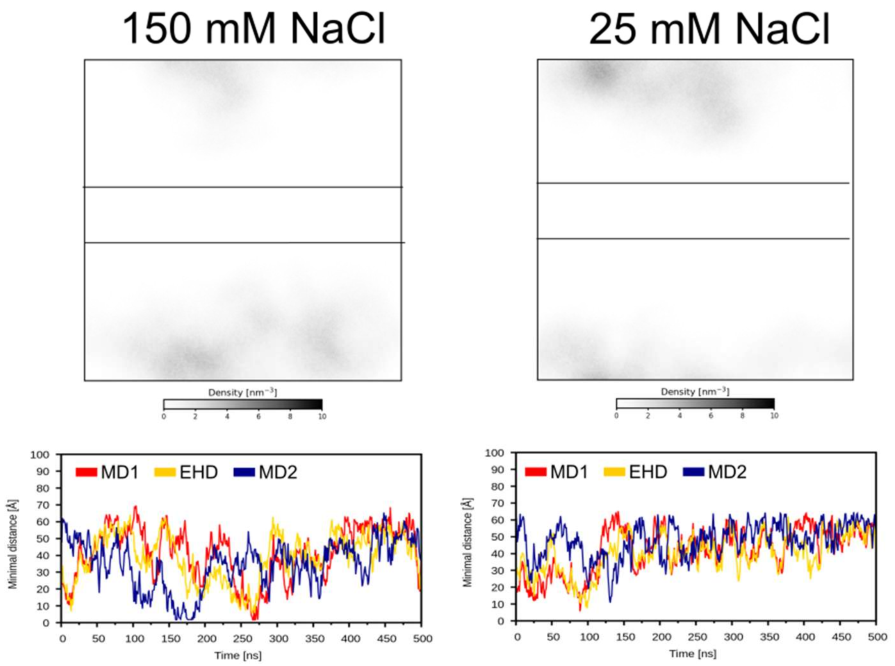

Figure S8. LipH^{Chap} localization along molecular dynamics simulations.

Top: Density maps of LipH^{Chap} localization within the simulation box calculated for 150 mM and 25 mM NaCl.

Bottom: Corresponding minimal distances of the individual LipH domains to the membrane over the course of each simulation.

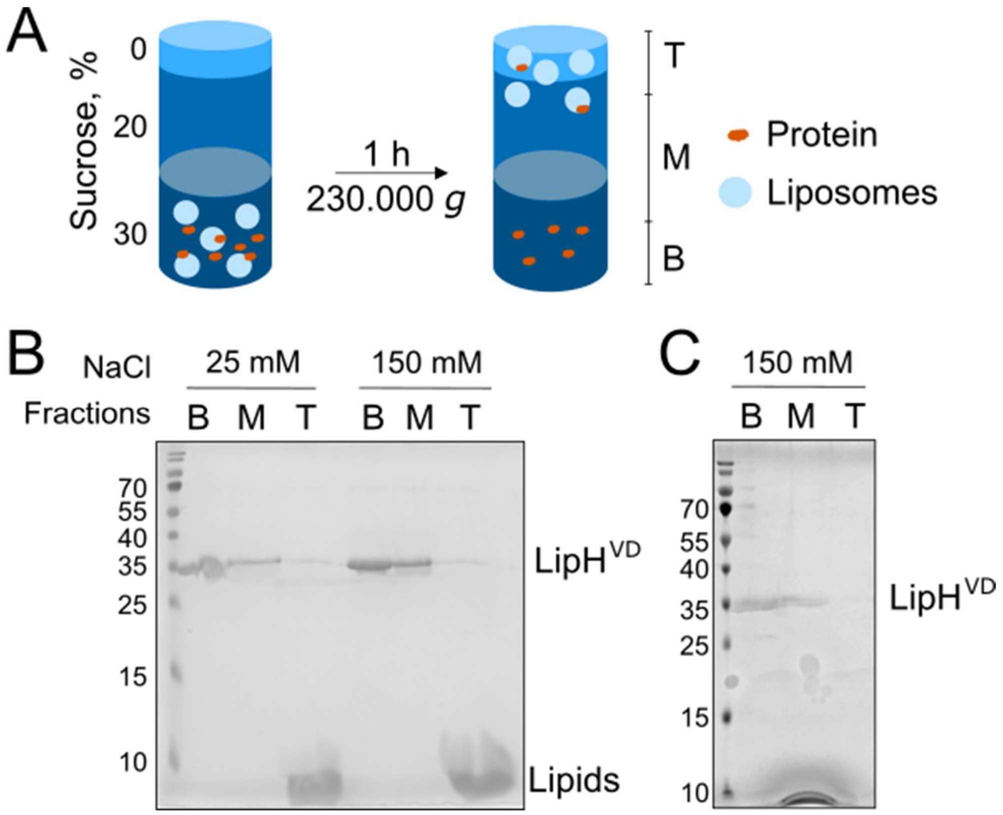

Figure S9. LipH^{VD}:membrane interactions at different ionic strengths.

(A) Schematics of the flotation assay to probe protein:membrane interactions. During centrifugation, the liposomes filled with a sucrose-free buffer migrate to the top of the sucrose density gradient. Liposome-bound proteins can afterwards be found in the top fraction (T) while the unbound and dissociated proteins predominantly stay in the bottom (B) and the middle fractions (M).

(B) SDS-PAGE of fractionated sucrose gradients at 25 mM and 150 mM mM NaCl in presence of liposomes composed of DOPC:DOPG. LipH^{VD} could be detected only in bottom and middle fractions, and the lipids could be seen in the top fraction.

(C) SDS-PAGE of the fractionated sucrose gradients 150 mM mM NaCl performed in absence of liposomes. LipH^{VD} could be detected only in bottom and middle fraction.

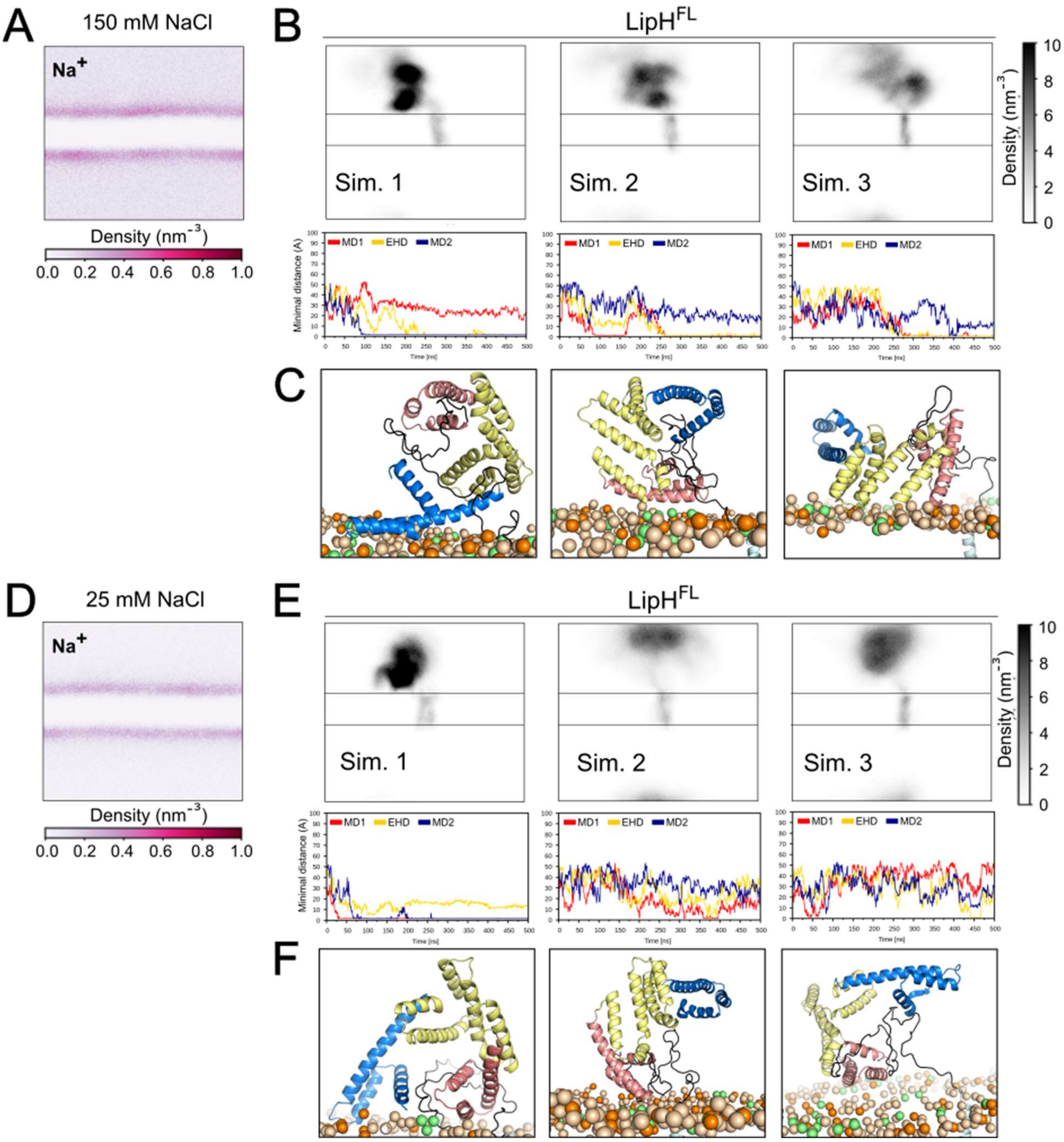

Figure S10. LipH interactions with the membrane interface.

(A) Exemplary density map of sodium ions at 150 mM NaCl (derived from simulation #1) highlights the lipid membrane interface.

(B) Density maps of LipH^{FL} localization within the simulation box calculated for individual simulations at 150 mM NaCl. The lipid bilayer borders are indicated as lines. Below: Corresponding minimal distances of the individual LipH domains to the membrane over the course of each simulation.

(C) A selection of poses taken by LipH at the membrane interface in simulations at 150 mM NaCl.

(D-F) Same as **(A-C)** upon simulations at 25 mM NaCl.

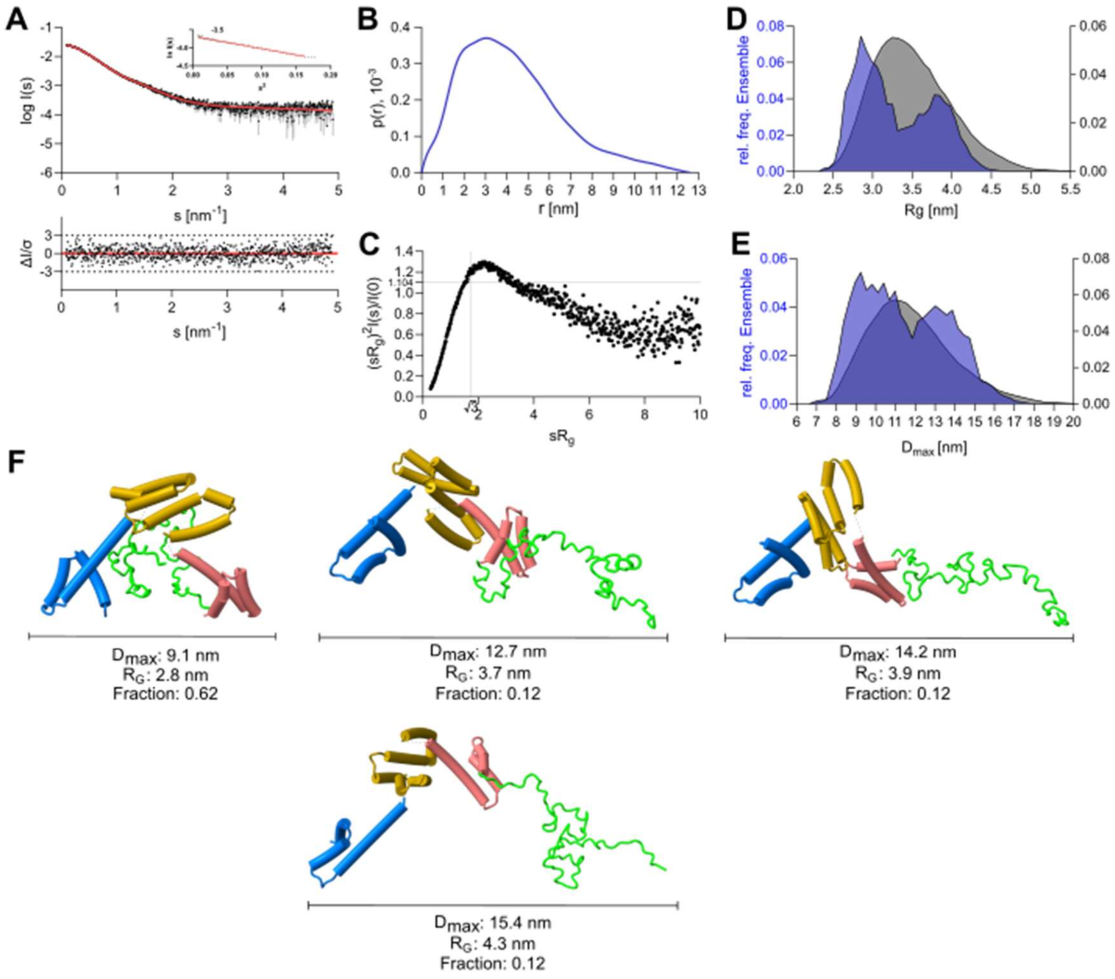

Figure S11. Small-angle X-ray scattering analysis of LipH^{VD}.

(A) Experimental data are shown in black dots, with grey error bars. The EOM model fit is shown as red line; below is the residual plot of the data. The Guinier plot of LipH^{VD} is shown in the inset.

(B) The pair distance distribution function *p(r)* of LipH^{VD} as determined by SAXS.

(C) Dimensionless Kratky plots of LipH^{VD}.

(D) Relative frequency distribution against the R_g of the EOM model. The selected ensemble is shown in blue, while the relative frequency of the initial pool is shown in grey.

(E) Relative frequency distribution of the D_{max} among the EOM model. The selected ensemble is shown in blue, while the relative frequency of the initial pool is shown in grey.

(F) Selected EOM models with their corresponding D_{max} and R_g values and the fractions within the ensemble.

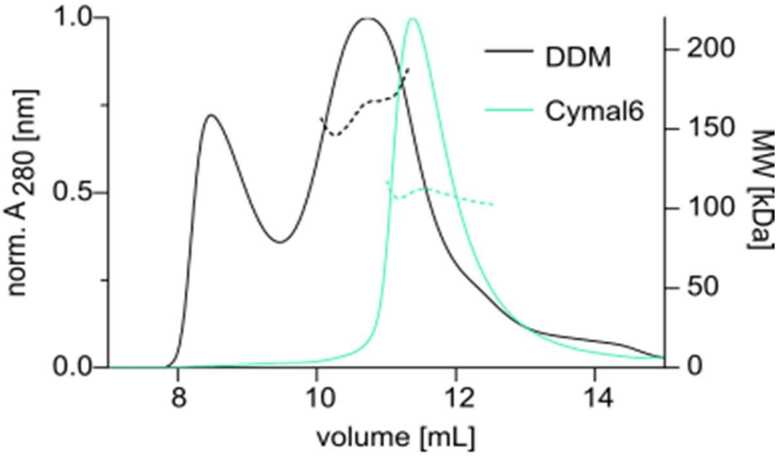

Figure S12. SEC-MALS of the full-length LipH in DDM and Cymal-6 micelles. Normalized UV absorbance of of LipH^{FL} in DDM (black) and Cymal-6 (petrol) is shown as solid lines. Dashed lines show the molecular weights in kDa determined by MALS (right Y-axis). Peak at ~8.5 mL for DDM-based sample likely reflected partial aggregation of the protein absence of glycerol, and it was excluded from analysis by fitting the chromatogram within the range of 10 to 12 mL.

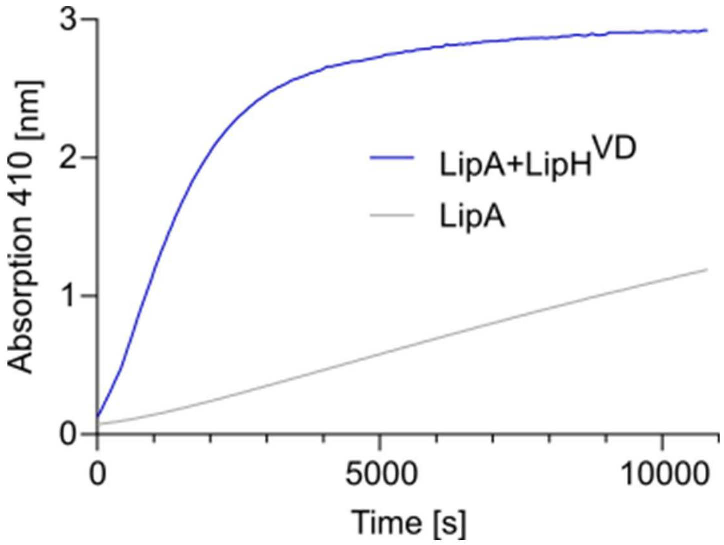

Figure S13: ***In vitro*** **analysis of LipH-mediated enzymatic activity of LipA.**

The lipase activity induced by LipH was monitored via hydrolysis of *p*-nitrophenyl butyrate to *p*-nitrophenolate and butyric acid, measured as an increase in absorbance at 410 nm. The substrate hydrolysis is shown in the presence of the chaperone LipH^{VD} (blue) and in its absence (grey).

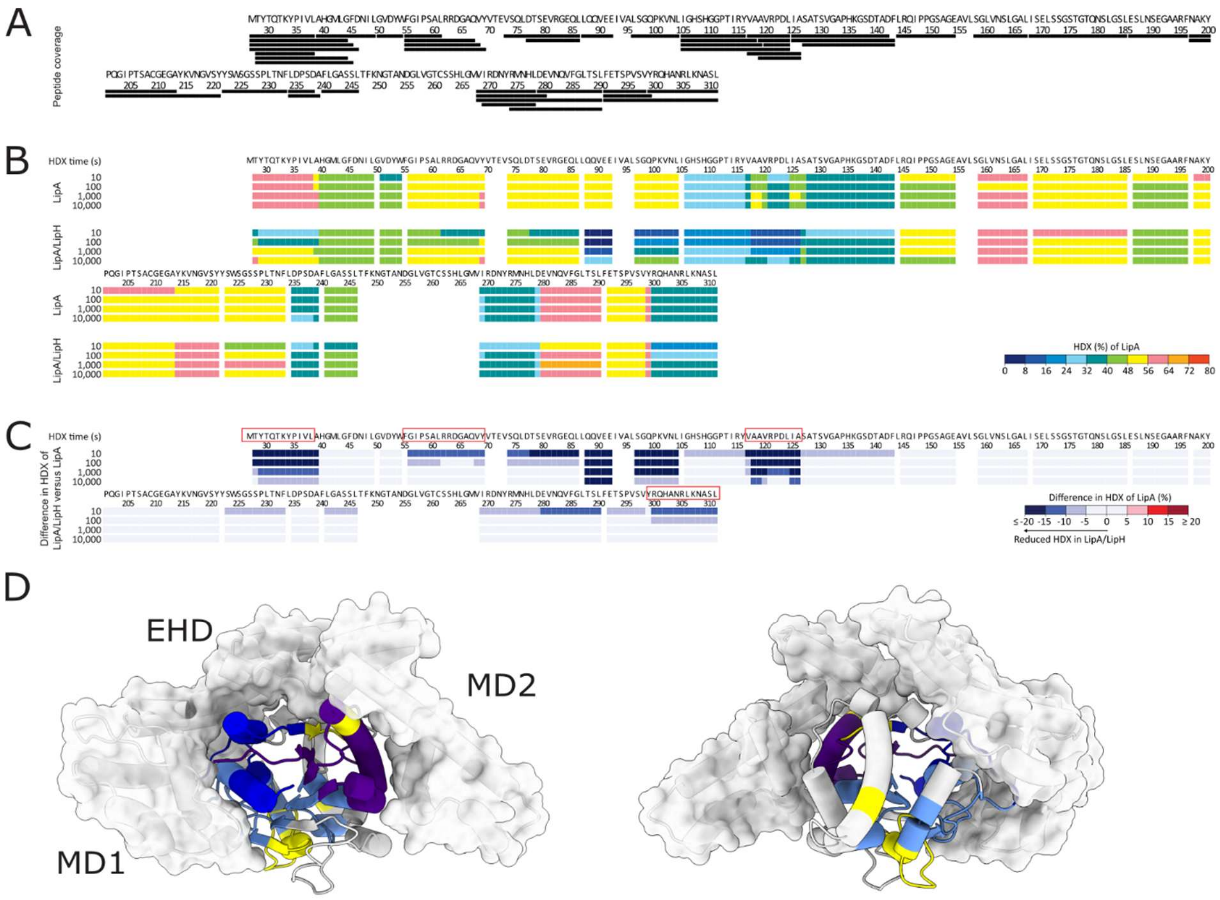

Figure S14. Hydrogen/deuterium exchange mass spectrometry of LipA.

(A) Overview of LipA peptides identified in HDX-MS experiments. Each black bar represents an identified peptide. In total, 49 peptides spanning 89.5% of the LipA^{F144E} amino acid sequence were analyzed for their H/D exchange.

(B) The residue-specific HDX of individual LipA and of LipA in presence of LipH^{Chap}, color-coded from 0% (blue) to 80% (red).

(C) The difference in the residue-specific HDX of LipA in presence of LipH^{Chap} and individual LipA is color-coded from ≤-20% (blue) to ≥20% (red). Red boxes indicate areas of bimodality in HDX (see also Suppl. Figure 15).

(D) Differences in the residue-specific HDX levels plotted on LipA structure in complex with LipH^{Chap}. The major HDX decrease is observed within the N-terminal fragment of LipA which interacts with MD2 of the chaperone.

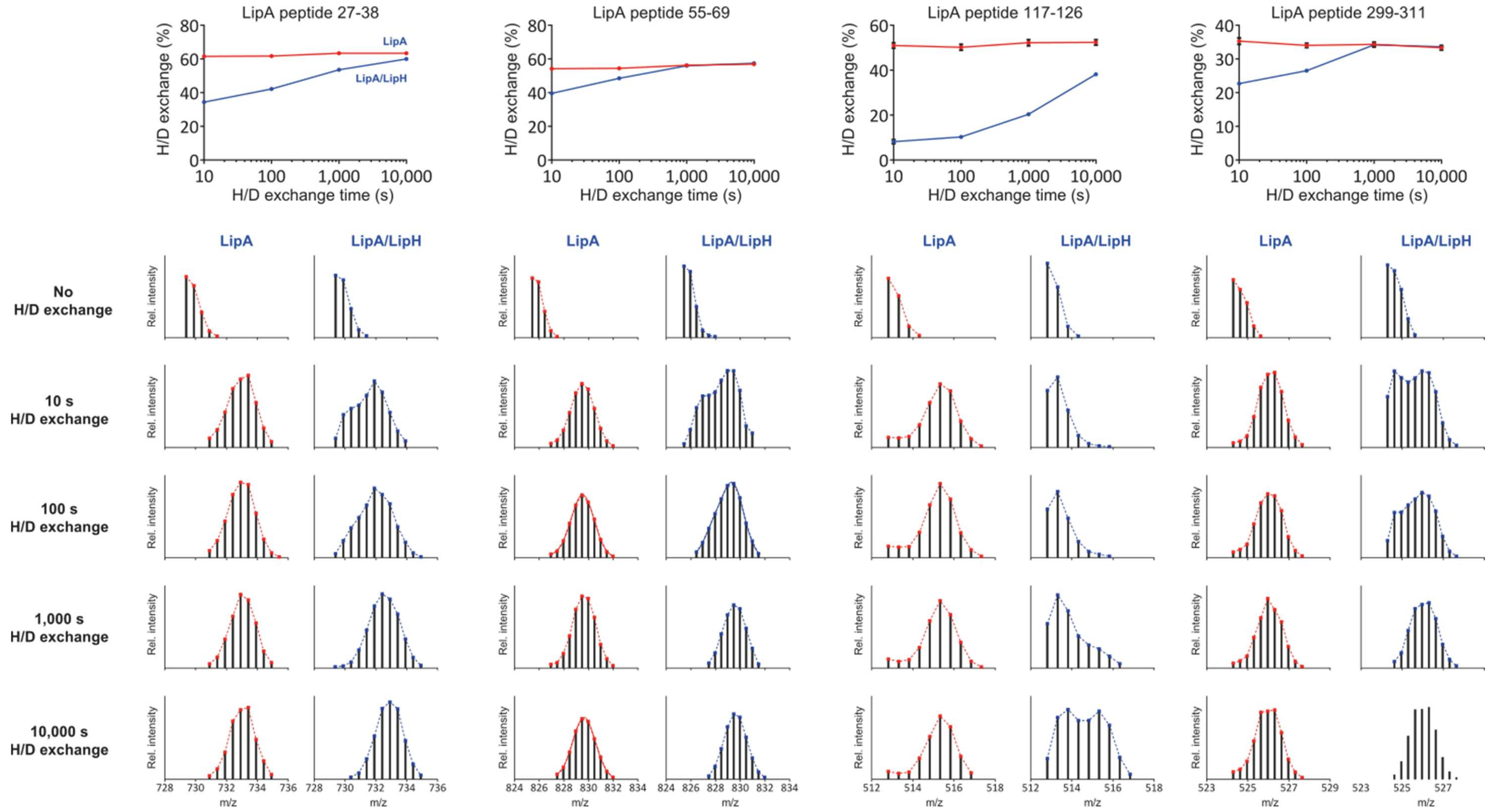

Figure S15. HDX behaviour of representative LipA peptides. *Top:* HDX over time observed for the indicated peptides of samples containing either LipA in isolation (red) or the LipA/LipH complex (blue). Data represent mean ± s.d. of n=3 technical replicates. *Bottom:* Mass spectra (depicted as ion sticks) of those representative LipA peptides over the HDX time course for either sample.

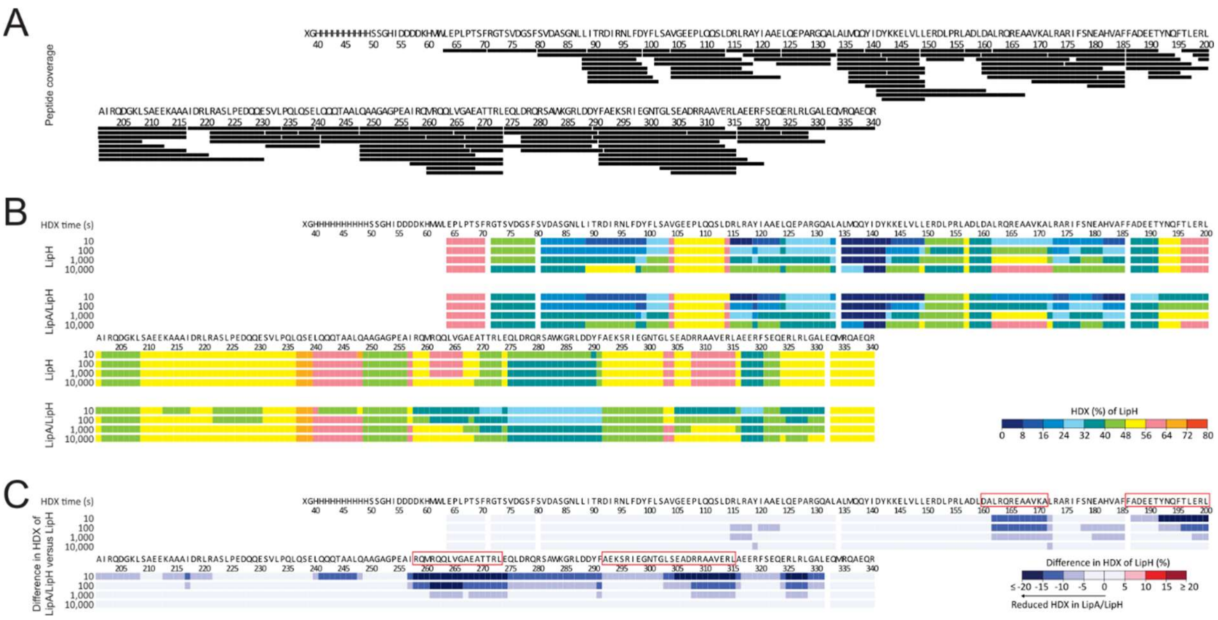

Figure S16. Hydrogen/deuterium exchange mass spectrometry of LipH^{Chap}.

(A) Overview of LipH^{Chap} peptides identified in HDX-MS experiments. Each black bar represents a peptide of LipH^{Chap}. In total, 126 peptides spanning 91.7% of the LipH^{Chap} amino acid sequence were analyzed for their H/D exchange.

(B) The residue-specific HDX of individual LipH^{Chap} and of LipH^{Chap} in presence of LipA is color-coded from 0% (blue) to 80% (red).

(C) The difference in residue-specific HDX of LipH^{Chap} in presence of LipA and individual LipH^{Chap} is color-coded from ≤-20% (blue) to ≥20% (red). Red boxes indicate areas of bimodality in HDX (see also Suppl. Figure 17).

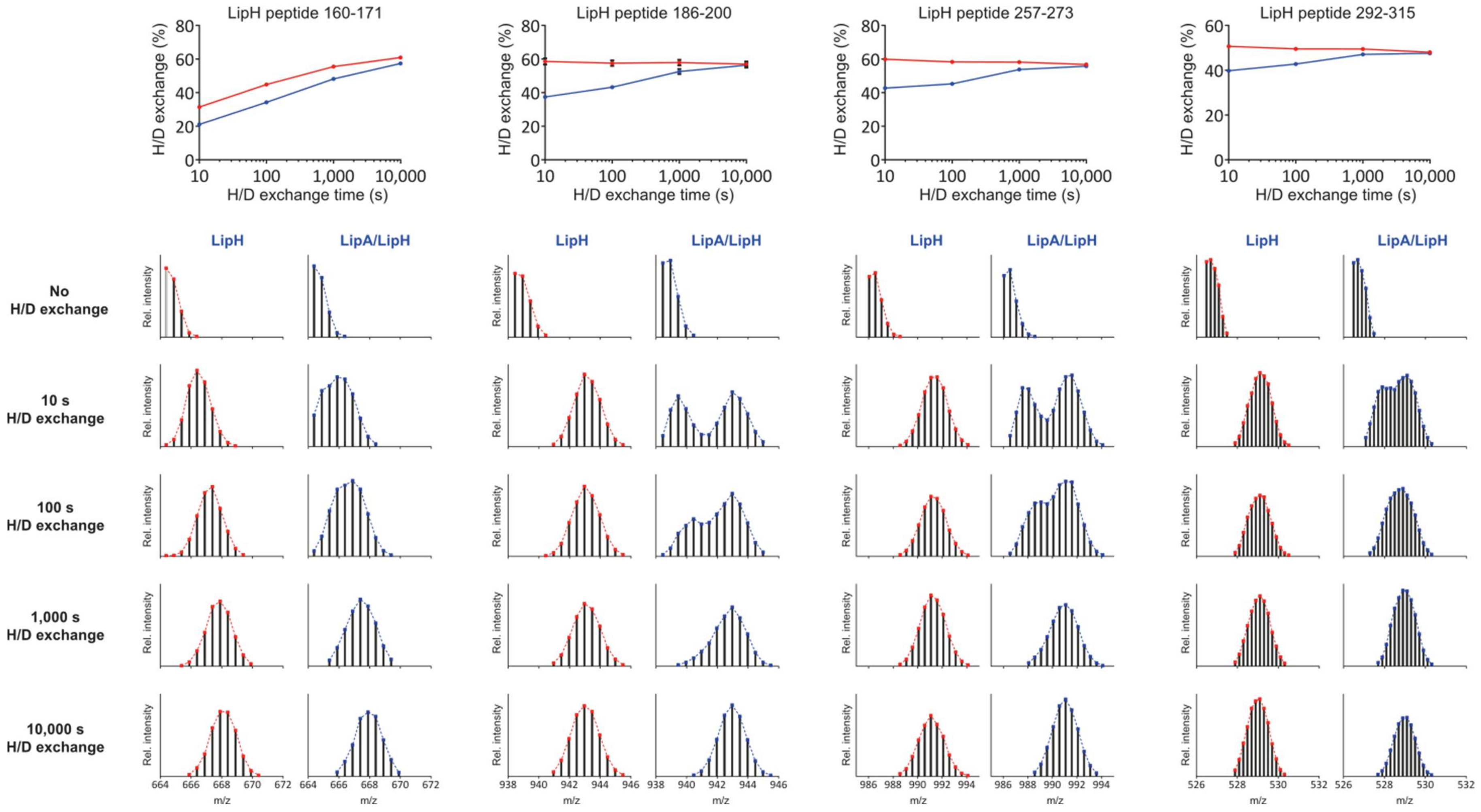

Figure S17. HDX behaviour of representative LipH^{Chap} peptides. *Top:* HDX over time observed for the indicated peptides of samples containing either LipH in isolation (red) or the LipA/LipH complex (blue). Data represent mean ± s.d. of n=3 technical replicates. *Bottom:* Mass spectra (depicted as ion sticks) of those representative LipH peptides over the HDX time course for either sample. For both LipA and LipH proteins two distinct species are apparent in the LipA/LipH complex samples from the mass spectra (**B**) likely due to an equilibrium between unbound and complex-bound LipA or LipH, respectively.

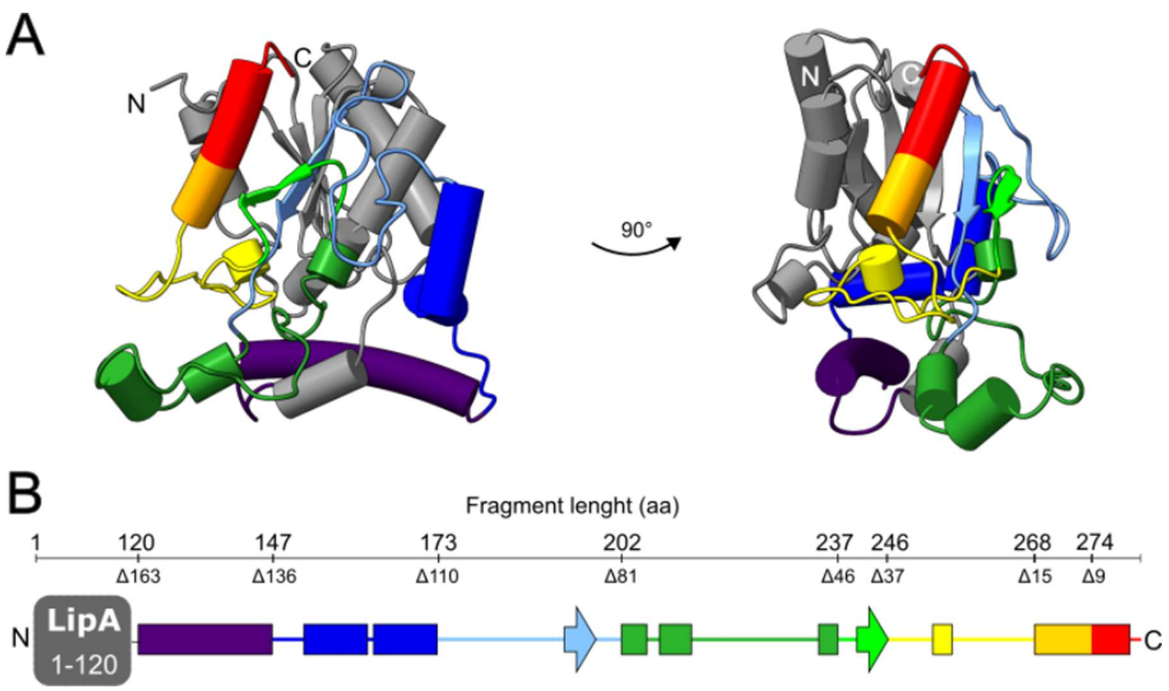

Figure S18. Design of LipA fragments for studying interactions with LipH.
(A) AlphaFold3 model of *P. aeruginosa* LipA with individual deletions highlighted using a rainbow color gradient (red to purple).

(B) Individual deletions were designed based on the secondary structure of LipA. α-Helices are indicated as bars, β-strands as arrows. The lengths of the designed fragments are indicated on the upper axis, the sizes of the corresponding C-terminal deletions are indicated below . The color-coding of the deleted fragments corresponds to that in the panel **(A)**.

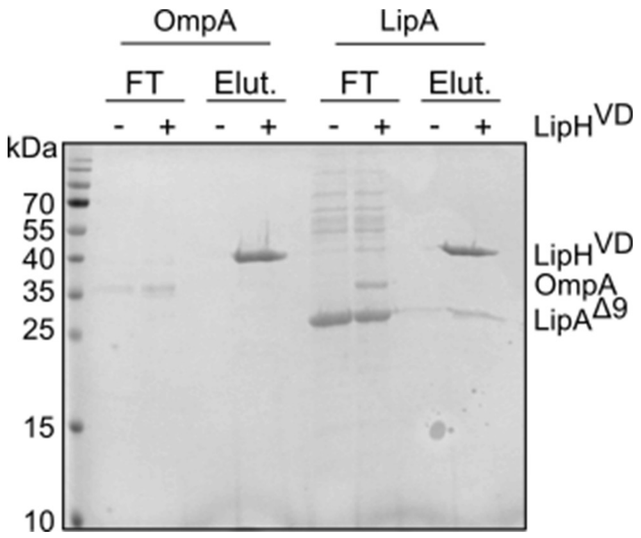

Figure S19. Co-elution assay to probe non-specific interactions of LipH.
SDS-PAGE of the assay to test unspecific interactions of LipH^{VD} using the outer membrane protein A (OmpA) of *E. coli* as a client. Elution fractions do not show a band for OmpA in presence or absence of LipH^{VD}, suggesting absence of interactions. The lipase fragment LipA$^{\Delta 9}$ is used as a positive reference.

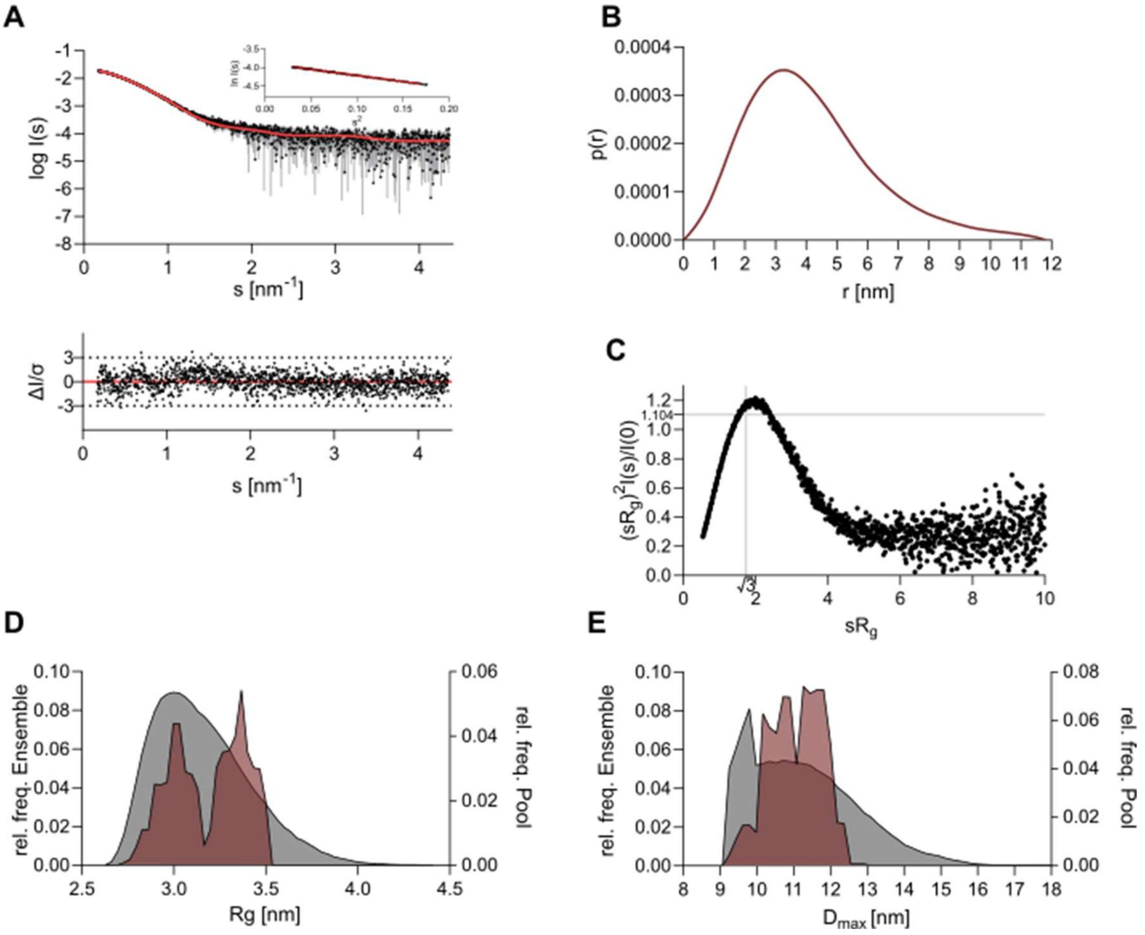

Figure S20. Small-angle X-ray scattering analysis of the LipH^{VD}:LipA complex.

(A) Experimental data are shown in black dots, with grey error bars. The EOM ensemble fit is shown as red line; below is the residual plot of the data. The Guinier plot of LipH^{VD}:LipA is shown in the inset.

(B) The pair distance distribution function p(r) of LipH^{VD}:LipA complex as determined by SAXS.

(C) Dimensionless Kratky plots of LipH^{VD}:LipA complex.

(D) Relative frequency distribution against the R_g of the EOM model. The ensemble is shown in brown, while the relative frequency of the pool is shown in grey.

(E) Relative frequency distribution against the D_{max} of the EOM model. The ensemble is shown in brown, while the relative frequency of the pool is shown in grey.

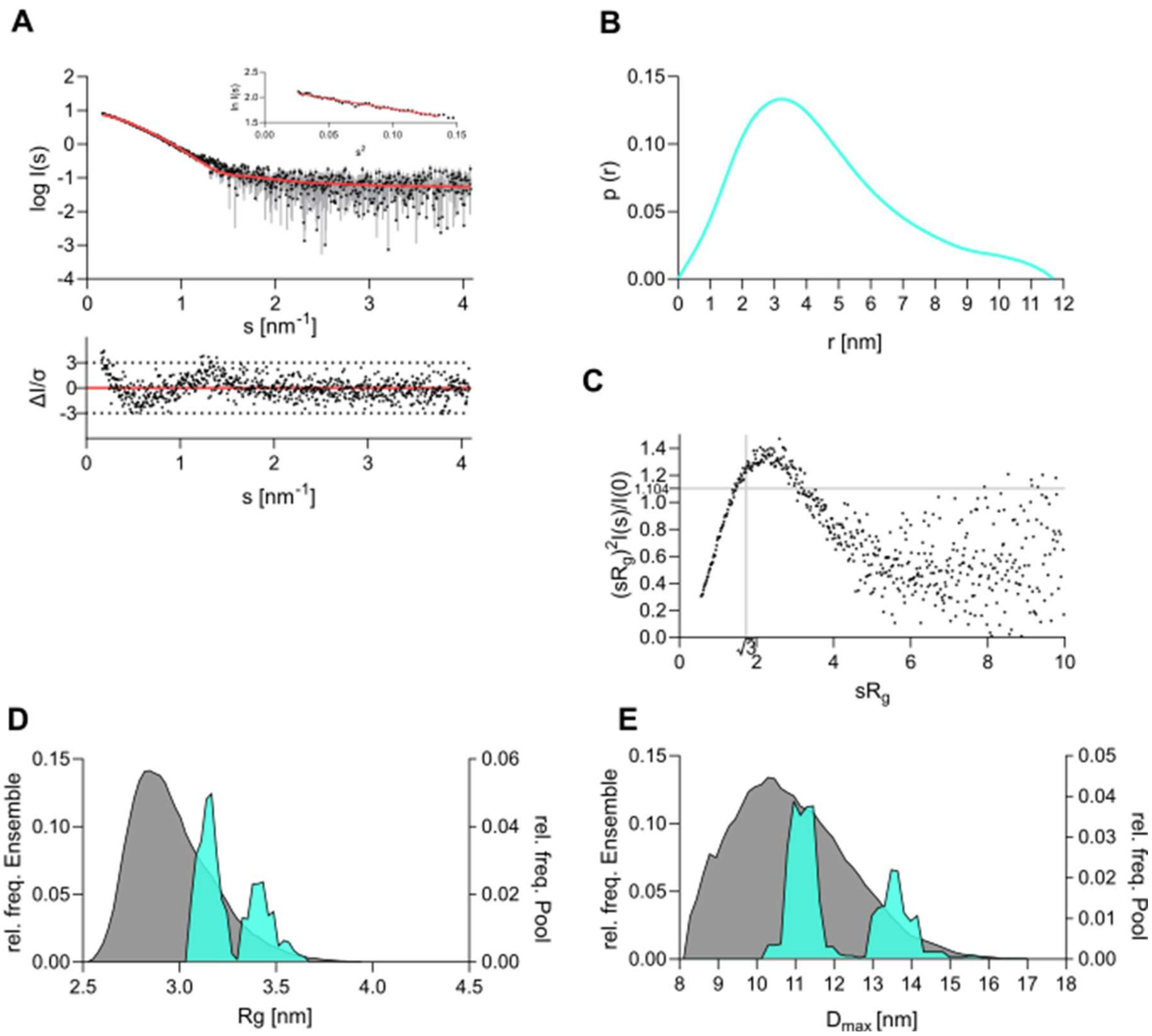

Figure S21. Small-angle X-ray scattering analysis of the LipH^{VD}:LipA$^{\Delta 81}$ complex.

(A) Experimental data are shown in black dots, with grey error bars. The EOM ensemble fit is shown as red line; below is the residual plot of the data. The Guinier plot of LipH^{VD}:LipA$^{\Delta 81}$ is shown in the inset.

(B) The pair distance distribution function *p(r)* of LipH^{VD}:LipA$^{\Delta 81}$ complex as determined by SAXS.

(C) Dimensionless Kratky plots of LipH^{VD}:LipA$^{\Delta 81}$ complex.

(D) Relative frequency distribution against the R_g in nm of the EOM model. The ensemble is shown in cyan, while the relative frequency of the pool is shown in grey.

(E) Relative frequency distribution against the D_{max} in nm of the EOM model. The ensemble is shown in cyan, while the relative frequency of the pool is shown in grey.

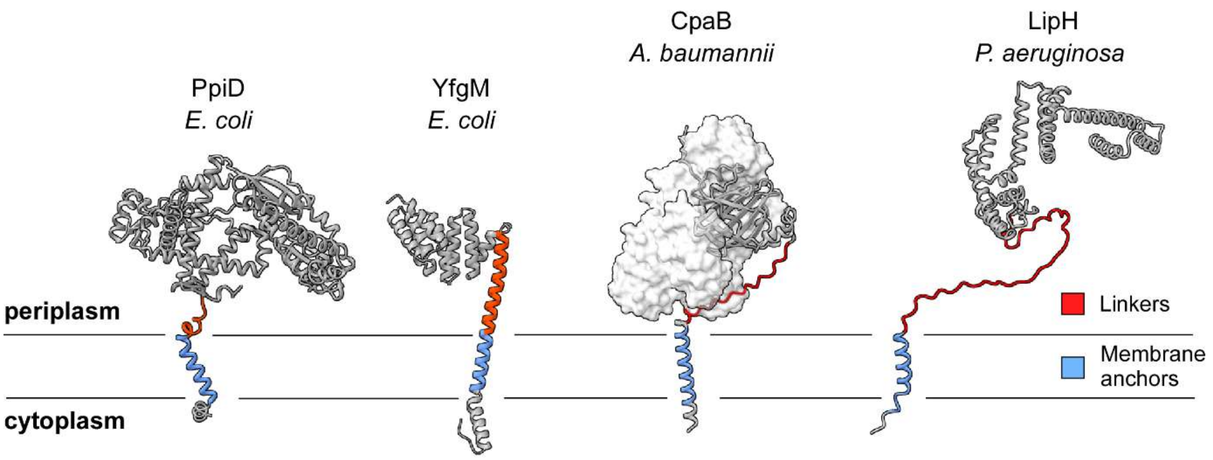

Figure S22. AlphaFod3-based models of the membrane-anchored chaperones. Membrane anchors are predicted based on the hydrophobicity via TMHMM. For *A. baumannii* CpaB chaperone, its cognate client protease CpaB is shown (surface visualization).

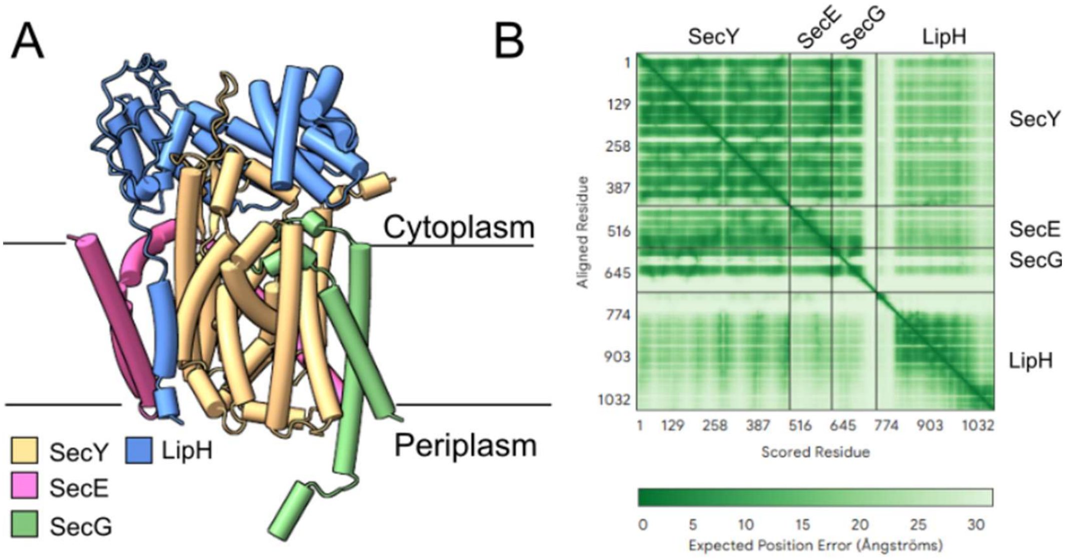

Figure S23. AlphaFold 3 analysis of a potential SecYEG:LipH^{FL} complex.

(A) AlphaFold3 model for the SecYEG:LipH^{FL} complex of *P. aeruginosa* (ipTM score 0.59).

(B) Predicted aligned error plot for the SecYEG:LipH^{FL} complex of *P. aeruginosa.*

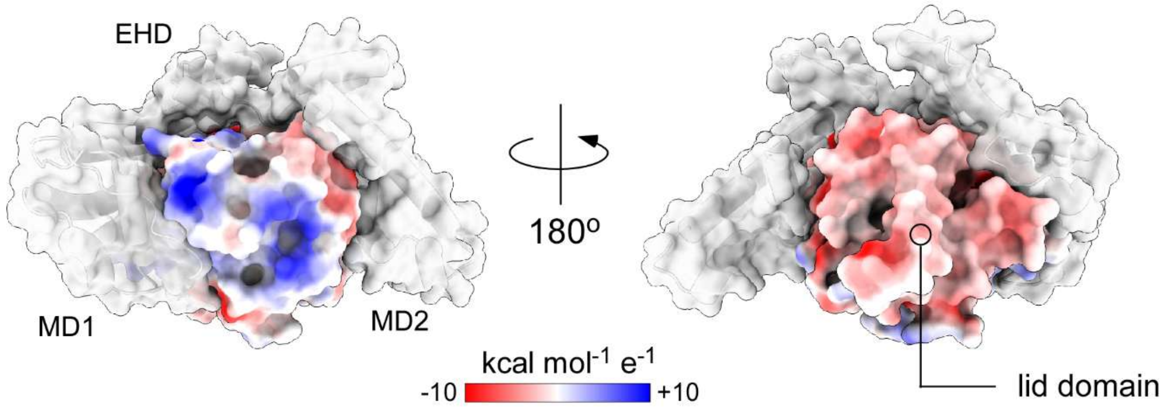

Figure S24. The electrostatic potential at the surface of the folded LipA. The molecular surface of LipA is coloured according to the local electrostatic potentials (blue = cationic; red = anionic; the scale bar shown below). The chaperoning domain of LipH is shown as a semi-transparent grey surface, with positions of the structural sub-domains indicated.

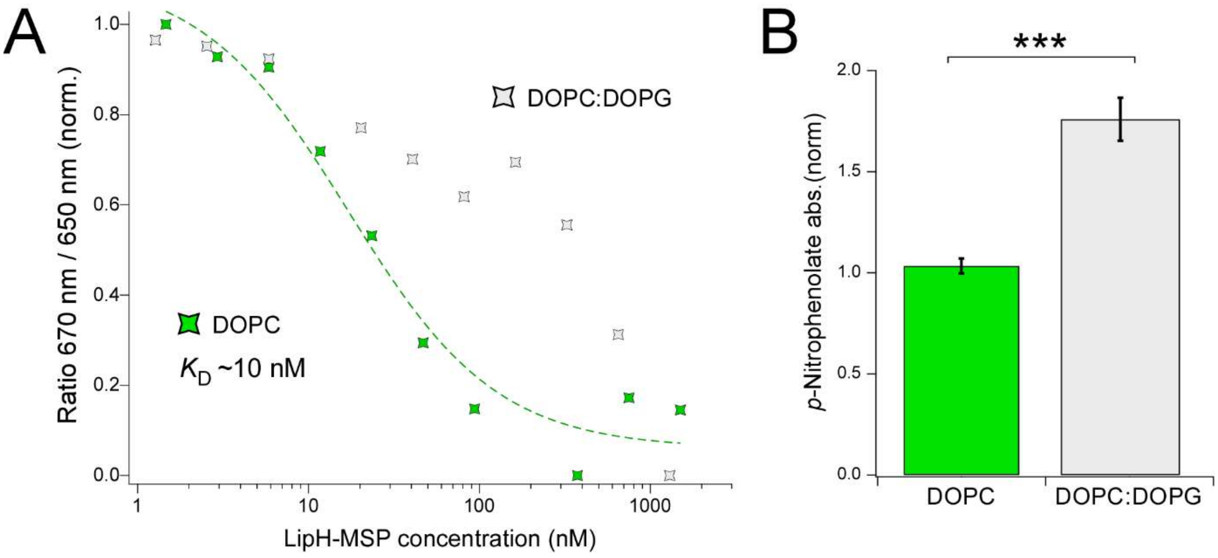

Figure S25. Lipid-dependent activity of LipH^{FL} in nanodiscs.

(A) Exemplary recordings of the spectral shift in LipA-CF647 fluorescence upon titrating LipH^{FL} reconstituted into nanodiscs with either DOPC or DOPC:DOPG lipids. Normalized ratios of LipA-CF647 fluorescence intensity at 670 nm and 650 nm are plotted against the LipH-MSP concentration. The apparent dissociation constant could be estimated for DOPC-based sample, but not for DOPC:DOPG sample due to the low affinity.

(B) Normalized lipase activity *in vitro* mediated by LipH^{FL} in presence of different lipids. Mean values are shown as bars, the error bars correspond to the standard deviations, calculated from three technical replicates. p-Value determined via one-way ANOVA test is 0.00036.

Table S1. Summary of SAXS data acquisition and analysis.

**Data collection parameters**			
SAXS Device	Xenocs Xeuss 2.0 with Q-Xoom	P12, PETRA III, DESY Hamburg (Blanchet et al. 2015)	BM29, ESRF Grenoble (Tully et al. 2023)
Detector	PILATUS 3 R 300K windowless	PILATUS 6 M	PILATUS3 x 2 M
Detector distance (m)	0.550	3.0	2.827
Beam size	0.8 mm x 0.8 mm	120 µm x 200 µm	200 µm x 100 µm
Wavelength (nm)	0.154	0.124	0.099
Sample environment	Low Noise Flow Cell, 1 mm ø	Quartz glass capillary, 1 mm ø	
Absolute scaling method	Comparison with scattering from pure H_2O		
Normalization	To transmitted intensity by beam-stop counter or direct beam		
Scattering intensity scale	Absolute scale, cm^{-1}		
s range (nm^{-1})‡	0.05 – 5.5	0.03 – 7.0	0.025–5.5
**Sample**	**LipH^{VD}**	**LipH^{VD}:LipA complex**	**LipH^{VD}:LipA$^{\Delta81}$ complex**
Organism	*Pseudomonas aeruginosa PAO1*		
UniProt ID	Q01725	LipH: Q01725<br>LipA: P26876	LipH: Q01725<br>LipA: P26876
Mode of measurement	batch	Online SEC-SAXS	
SEC-Column	-	Superdex 200 Increase 10/300 GL	
Flowrate (mL/min)	-	0.6	0.6
Injection volume (µL)	-	100	100
Temperature (°C)	10	20	20
Exposure time s (# frames)	600 (24)	0.995 (2400)	2 (1200)
# frames used for averaging	20	24	18
Protein buffer	50 mM Tris, 100 mM NaCl, 100 µM TCEP, 5% glycerol, pH 8.0	5 mM Tris, 5 mM glycine, 1 mM $CaCl_2$, 5% glycerol, pH 9.0	5 mM Tris, 5 mM glycine, 1 mM $CaCl_2$, 5% glycerol, pH 9.0
Protein concentration [mg/mL]	11.82	8 mg/mL LipH^{VD} + 0.8 mg/mL LipA^{FL}	1 mg/mL LipH^{VD} + LipA^{d81}
**Structural parameters**			
Guinier Analysis (PRIMUS)			
I(0) ± s (cm^{-1})	0.025 ± 0.0001	0.021 ± 0.00004	8.834 ± 0.072
R_g ± s (nm)	3.22 ± 0.022	3.14 ± 0.001	3.51 ± 0.043
s-range (nm^{-1})	0.088 – 0.403	0.173 – 0.411	0.163 – 0.369
min < sRg < max limit	0.282 – 1.297	0.544 – 1.289	0.572 – 1.294
Data point range	1 – 55	1 – 85	1 – 43
Linear fit assessment (R^2)	0.990	0.996	0.972

PDDF/P® Analysis (GNOM)			
$I(0) \pm s$ (cm^{-1})	0.026 ±0.0001	0.021 ± 0.00003	8.874 ± 0.058
$R_g \pm s$ (nm)	3.41 ± 0.026	3.26 ± 0.0095	3.53 ± 0.028
r_{max} (nm)	12.61	11.82	11.70
Porod volume (nm^3)	69.97	110.09	103.55
s-range (nm^{-1})	0.088 – 4.895	0.173 – 4.360	0.163 – 4.081
χ2 / CorMap P-value	1.015 / 0.553	1.003 / 0.087	1.069 / 0.092
**Molecular mass (kDa)**			
From *I*(0)	34.62	n.d.	n.d.
From Qp (Porod 1951)	40.03	69.34	63.04
From MoW2 (Fischer et al. 2010)	34.20	68.07	51.40
From Vc (Rambo and Tainer 2013)	38.87	66.58	55.40
Bayesian Inference (Hajizadeh et al. 2018)	37.70	67.08	58.15
From sequence	38.32	68.46 (1:1 stoichiometry)	59.61 (1:1 stoichiometry)
**Flexibility ensemble modelling**			
EOM			
Symmetry	P1	P1	P1
s-range for fit (nm^{-1})	0.088 – 4.895	0.173 – 4.360	0.163 – 4.081
χ^2, CorMap *P*-value	1.026 / 0.553	1.136 / 0.166	1.528 / 0. 000003
**SASBDB accession codes** (Kikhney et al. 2020)	SASDYM9	SASDYN9	SASDYP9
**Software**			
ATSAS Software Version (Manalastas-Cantos et al. 2021)	3.0.5		
Primary data reduction	CHROMIXS (Panjkovich and Svergun 2018)/ PRIMUS (Konarev et al. 2003)		
Data processing	GNOM (Svergun 1992)		
Flexibility ensemble modellin	EOM (Bernado et al. 2007; Tria et al. 2015)		
Statistic goodness-of-fit test	χ 2, CorMap (Franke, Jeffries, and Svergun 2015)		
Model visualization	Chimera X 1.10 (Goddard et al. 2018)		

‡s = 4πsin(θ)/λ, 2θ – scattering angle, n.d. not determined

Table S2. Overview of data obtained by hydrogen/deuterium exchange mass spectrometry.

Protein	$LipA^{F144E}$	$LipH^{Chap}$
Conditions of H/D exchange	25 °C in 5 mM Tris-HCl pH 9.0, 5 mM glycine, 1 mM $CaCl_2$<br>Final D_2O during HDX = 89.5% (v/v)	
Time course of H/D exchange	10/100/1,000/10,000 s	
Samples	1) 50 µM $LipA^{F144E}$ (5 µM during HDX)<br>2) 50 µM $LipH^{Chap}$ (5 µM during HDX)<br>3) 50 µM $LipA^{F144E}$ + 50 µM $LipH^{Chap}$ (5 µM during HDX)	
Replicates	3 technical replicates (separate H/D exchange reactions)	
Number of Peptides	49	126
Average peptide length (aa)	10.86	11.44
Sequence coverage (%)	89.5	91.7
Redundancy	2.47	6.09
Back-exchange	No correction for back-exchange	
Repeatability (average SD)	0.06 Da / 0.47%	0.07 Da / 0.62%
Significance criterium applied	5% difference in relative HDX	

Supplemental references

Bernado, P., E. Mylonas, M. V. Petoukhov, M. Blackledge, and D. I. Svergun. 2007. 'Structural characterization of flexible proteins using small-angle X-ray scattering', *J Am Chem Soc*, 129: 5656-64.

Blanchet, C. E., A. Spilotros, F. Schwemmer, M. A. Graewert, A. Kikhney, C. M. Jeffries, D. Franke, D. Mark, R. Zengerle, F. Cipriani, S. Fiedler, M. Roessle, and D. I. Svergun. 2015. 'Versatile sample environments and automation for biological solution X-ray scattering experiments at the P12 beamline (PETRA III, DESY)', *J Appl Crystallogr*, 48: 431-43.

Fischer, H., M. D. Neto, H. B. Napolitano, I. Polikarpov, and A. F. Craievich. 2010. 'Determination of the molecular weight of proteins in solution from a single small-angle X-ray scattering measurement on a relative scale', *Journal of Applied Crystallography*, 43: 101-09.

Franke, D., C. M. Jeffries, and D. I. Svergun. 2015. 'Correlation Map, a goodness-of-fit test for one-dimensional X-ray scattering spectra', *Nat Methods*, 12: 419-22.

Goddard, T. D., C. C. Huang, E. C. Meng, E. F. Pettersen, G. S. Couch, J. H. Morris, and T. E. Ferrin. 2018. 'UCSF ChimeraX: Meeting modern challenges in visualization and analysis', *Protein Sci*, 27: 14-25.

Hajizadeh, N. R., D. Franke, C. M. Jeffries, and D. I. Svergun. 2018. 'Consensus Bayesian assessment of protein molecular mass from solution X-ray scattering data', *Sci Rep*, 8: 7204.

Kikhney, A. G., C. R. Borges, D. S. Molodenskiy, C. M. Jeffries, and D. I. Svergun. 2020. 'SASBDB: Towards an automatically curated and validated repository for biological scattering data', *Protein Sci*, 29: 66-75.

Konarev, P. V., V. V. Volkov, A. V. Sokolova, M. H. J. Koch, and D. I. Svergun. 2003. ': a Windows PC-based system for small-angle scattering data analysis', *J Appl Crystallogr*, 36: 1277-82.

Manalastas-Cantos, K., P. V. Konarev, N. R. Hajizadeh, A. G. Kikhney, M. V. Petoukhov, D. S. Molodenskiy, A. Panjkovich, H. D. T. Mertens, A. Gruzinov, C. Borges, C. M. Jeffries, D. I. Svergun, and D. Franke. 2021. 'ATSAS 3.0: expanded functionality and new tools for small-angle scattering data analysis', *J Appl Crystallogr*, 54: 343-55.

Panjkovich, A., and D. I. Svergun. 2018. 'CHROMIXS: automatic and interactive analysis of chromatography-coupled small-angle X-ray scattering data', *Bioinformatics*, 34: 1944-46.

Porod, G. 1951. 'Die Rontgenkleinwinkelstreuung Von Dichtgepackten Kolloiden Systemen .1.', *Kolloid-Zeitschrift and Zeitschrift Fur Polymere*, 124: 83-114.

Rambo, R. P., and J. A. Tainer. 2013. 'Accurate assessment of mass, models and resolution by small-angle scattering', *Nature*, 496: 477-81.

Svergun, D. I. 1992. 'Determination of the Regularization Parameter in Indirect-Transform Methods Using Perceptual Criteria', *J Appl Crystallogr*, 25: 495-503.

Tria, G., H. D. Mertens, M. Kachala, and D. I. Svergun. 2015. 'Advanced ensemble modelling of flexible macromolecules using X-ray solution scattering', *Iucrj*, 2: 207-17.

Tully, M. D., J. Kieffer, M. E. Brennich, R. Cohen Aberdam, J. B. Florial, S. Hutin, M. Oscarsson, A. Beteva, A. Popov, D. Moussaoui, P. Theveneau, G. Papp, J. Gigmes, F. Cipriani, A. McCarthy, C. Zubieta, C. Mueller-Dieckmann, G. Leonard, and P. Pernot. 2023. 'BioSAXS at European Synchrotron Radiation Facility - Extremely Brilliant Source: BM29 with an upgraded source, detector, robot, sample environment, data collection and analysis software', *J Synchrotron Radiat*, 30: 258-66.
